# Prevalence, concordance and determinants of human papillomavirus infection among heterosexual partners in a rural region in central Mexico

**DOI:** 10.1186/1471-2334-11-25

**Published:** 2011-01-26

**Authors:** Rocio Parada, Rosalba Morales, Anna R Giuliano, Aurelio Cruz, Xavier Castellsague, Eduardo Lazcano-Ponce

**Affiliations:** 1 Centro de Investigación en Salud Poblacional, Instituto Nacional de Salud Pública, Cuernavaca, Morelos, México; 2 Instituto Mexicano del Seguro Social, Morelos, México; 3 H. Lee Moffitt Cancer Center and Research Institute, Tampa, Florida; 4 Cancer Epidemiology Research Program, Institut Català d'Oncologia (ICO), IDIBELL, CIBER-ESP, L'Hospitalet de Llobregat, Spain

## 

Following the publication of this paper[1] we received some important observations on the statistical proof used and the way the results were presented in the tables and figure. We have taken them into account and are responding to the same.

For the comparison of the prevalence of HPV infection in men and women, we used the MacNemar test. This test is used to prove a hypothesis of equality of proportions in non-independent groups. In this case the groups of men and women are not independent because they are sexual partners. Table 1 shows that the prevalence of HPV is greater in men than in women (20.4% vs 13.7%, p value = 0.0009). There were no statistically significant differences between type specific infection in men and women; only in types HPV31, HPV53, HPV55, HPV61 and HPV84 (Table 1 and Figure 1).

**Table 1:** Prevalence of HPV DNA in 504 heterosexual couples in central Mexico, according to sex

	Men n = 504		Women n = 504				
	
HPV	n	%	n	%	OR*	CI 95%*	ρ*
**Presence of HPV**							
Positive	103	20.4	69	13.7	0.51	(0.33-0.77)	0.0009
**Presence of high-risk HPV**							
Positive	44	8.7	48	9.5	1.14	(0.67-2.00)	0.6056
**Presence of low-risk HPV**							
Positive	75	14.9	33	6.5	0.27	(0.15-0.49)	0.0000
**Multiple HPV infection**							
One type only	79	15.7	50	9.9			
Two or more types	24	4.8	19	3.8	0.74	(0.34-1.55)	0.3841
**Presence of HPV 16 and/or 18**							
Negative	491	97.4	490	97.2			
Positive	13	2.6	14	2.8	1.09	(0.44-2.72)	0.8348
							
**Positive for**							
**High-risk HPV**							
16	6	1.2	10	2	1.80	(0.54-6.83)	0.2850
18	7	1.4	4	0.8	0.50	(0.08-2.34)	0.3173
31	1	0.2	5	1			0.0455
33	0	0	0	0			
35	0	0	0	0			
39	7	1.4	3	0.6	0.20	(0.01-1.78)	0.1025
45	2	0.4	1	0.2	0.50	(0.01-9.60)	0.5637
51	2	0.4	3	0.6	1.50	(0.17-17.96)	0.6547
52	3	0.6	5	1	2.00	(0.29-22.10)	0.4142
56	2	0.4	1	0.2	0.00	(0.00-39.00)	0.3173
58	3	0.6	5	1	2.00	(0.29-22.10)	0.4142
59	12	2.4	15	3	1.37	(0.50-3.93)	0.4913
66	6	1.2	3	0.6	0.40	(0.04-2.44)	0.2568
**For low-risk HPV**							
6	2	0.4	2	0.4	1.00	(0.01-78.40)	1.0000
11	0	0	0	0			
26	0	0	0	0			
40	2	0.4	2	0.4	1.00	(0.07-13.70)	1.0000
42	2	0.4	2	0.4	1.00	(0.07-13.70)	1.0000
53	10	2	2	0.4	0.11	(0.01-0.80)	0.0114
54	5	1	4	0.8	0.66	(0.05-5.81)	0.6547
55	4	0.8	0	0	0.00	(0.00-1.51)	0.0450
61	14	2.8	2	0.4	0.07	(0.01-0.51)	0.0013
62	11	2.2	7	1.4	0.43	(0.07-1.87)	0.2059
64	0	0	0	0			
67	0	0	0	0			
68	2	0.4	1	0.2	0.50	(0.01-9.60)	0.5637
69	0	0	1	0.2			0.3173
70	1	0.2	0	0	0.00	(0.00-39.00)	0.3171
71	3	0.6	5	1	2.00	(0.29-22.10)	0.4142
72	4	0.8	1	0.2	0.25	(0.01-2.52)	0.1797
73	2	0.4	2	0.4	1.00	(0.07-13.79)	1.0000
81	7	1.4	4	0.8	0.50	(0.08-2.34)	0.3173
82	0	0	0	0			
83	1	0.2	2	0.4	2.00	(0.10-117.90)	0.5637
84	9	1.8	1	0.2	0.00	(0.00-0.58)	0.0047
IS39	0	0	0	0			
Cp6108	5	1	3	0.6	0.50	(0.05-3.48)	0.4142

* OR, CI95% and p-value obtained using McNemar's Test.

**Figure 1:**
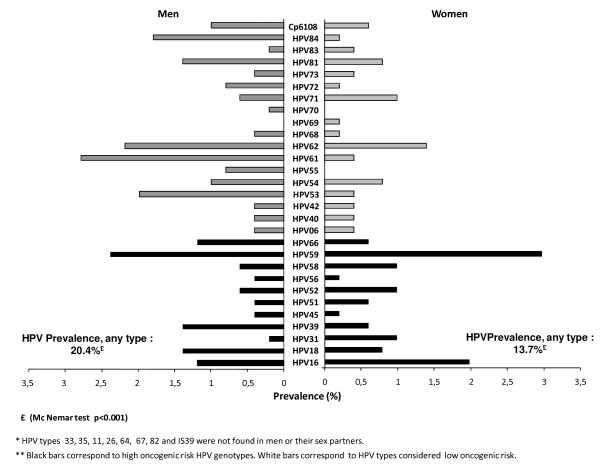
Type specific prevalence of HPV infection in a group of heterosexual couples in central Mexico, according to sex

The analysis of known risk factors for HPV infection was carried out separately for men and women. Non-conditional logistic regression was performed. When stratifying by sex we do not need to consider the condition of sexual partners. This part of the analysis was performed in this way, as it allows us to include explanatory variables in men - variables that cannot be defined in women, such as circumcision, use of condoms, and some specific characteristics on sexual risk behaviors. In women it allows us to consider, in addition to characteristics of their own sexual behaviors, characteristics of their male partner's sexual behavior - circumcision, use of condoms, etc. (Table 2). The last section of the study focuses on assessing the risk of HPV infection in women, considering the presence of HPV infection in their sex partners as an explanatory variable. Thus we find that women whose sexual partners are HPV positive have 5.15 times greater risk of HPV, compared to those whose partners are HPV negative (CI 95% 3.01, 8.82). Indeed, what matters to us in this part is proving that the variable "presence of HPV in male partner" be associated with the presence of HPV in the female. We do not seek to compare the risk of HPV infection between men and women (Table 3).

**Table 2:** Sociodemographic and sexual conduct characteristics associated with the presence of HPV DNA among 504 heterosexual couples in central Mexico, according to sex

	Men **n = 504**^**a**^				Women **n = 504**^**a**^			
Variable		HPV + n = 103	Risk of HPV infection			HPV + n = 69	Risk of HPV infection	
	**n (%)**	**HPV + (%)**	**OR**^**b**^	**CI 95%**	**n (%)**	**HPV + (%)**	**OR**^**b**^	**CI 95%**

**Age**^**c **^**(years)**								
18-24	40(8.0)	9(22.5)	1.00		64(12.7)	13(20.3)	1.00	
25-30	91(18.0)	17(18.7)		(0.31-1.93)	98(19.4)	15(15.3)		(0.30-1.60)
31-40	191(37.9)	29(15.2)	0.77 0.61	(0.26-1.42)	209(41.5)	24(11.5)	0.70 0.47	(0.22-1.00)
41-75	182(36.1)	48(26.4)	1.23	(0.54-2.80)	133(26.4)	17(12.8)	0.55	(0.24-1.23)
*p-trend*				0.1999				0.1305
**Place of residence**								
Rural	350(69.4)	62(17.7)	1.00		350(69.4)	47(13.4)	1.00	
Urban	154(30.6)	41(26.6)	1.71	(1.08-2.71)	154(30.6)	22(14.3)	1.02	(0.58-1.79)
**Marital Status**								
Married	400(79.4)	72(18.0)	1.00		400(79.4)	43(10.7)	1.00	
Single	104(20.6)	31(29.8)	1.92	(1.14-3.25)	104(20.6)	26(25.0)	2.79	(1.56-5.00)
**Schooling**^**d**^								
< = 6 years	174(34.5)	47(27.0)	1.85	(0.99-3.44)	77(15.5)	8(10.4)	0.70	(0.28-1.76)
7-9 years	199(39.5)	37(18.6)	1.28		286(57.6)	43(15.0)	1.17	(0.62-2.19)
> = 10 years	131(26.0)	19(14.5)	1.00	(0.70-2.36)	134(26.9)	17(12.7)	1.00	
*p-trend*				0.0061				0.8069
**Religion**								
Catholic	430(85.3)	81(18.8)	1.00		430(85.3)	58(13.5)	1.00	
Other	74(14.7)	22(29.7)	1.88	(1.07-3.31)	74(14.7)	11(14.9)	1.04	(0.51-2.11)
**Current smoker**								
No	278(55.2)	56(20.1)	1.00		435(86.3)	53(12.2)	1.00	
Yes	226(44.8)	47(20.8)	1.08	(0.69-1.69)	69(13.7)	16(23.2)	1.97	(1.03-3.75)
**Age on initiating sexual life**								
≤18 years	284(56.3)	68(23.9)	1.59	(1.00-2.52)	269(53.4)	39(14.5)	1.06	(0.62-1.81)
≥19 years	220(43.7)	35(15.9)	1.00		235(46.6)	30(12.8)	1.00	
**No. of lifetime sexual partners**								
One	185(36.7)	30(16.2)	1.00		371(73.6)	45(12.1)	1.00	
Two	76(15.1)	17(22.4)		(0.75-2.92)	88(17.5)	15(17.1)		(0.78-2.85)
Three to nine	171(33.9)	31(18.1)	1.49	(0.62-1.90)	45(8.9)	9(20.0)	1.50	(0.75-3.79)
Ten or more	72(14.3)	25(34.7)	1.08	(1.34-4.82)	--	--	1.69	--
			2.54				--	
*P-trend*				0.0142				0.0796
**History of anal sexual relations**								
No	305(63.1)	64(20.9)	1.00		146(67.0)	25(17.1)	1.00	
Yes	178(36.9)	34(19.1)	0.90	(0.56-1.45)	72(33.0)	8(11.1)	0.65	(0.26-1.60)
**Circumcision**^**e**^								
No	469(93.0)	98(20.9)	1.00		469(93.0)	61(13.0)	1.00	
Yes	35(7.0)	5(14.3)	0.61	(0.22-1.64)	35(7.0)	8(22.9)	1.92	(0.82-4.51)
**History of sexual relations with prostitutes**								
No	395(78.4)	72(18.2)	1.00		--	--	--	
Yes	109(21.6)	31(28.4)	1.68	(1.01-2.78)	--	--	--	--
**Use of condom when having sexual relations with prostitutes**								
Have not had sexual relations with prostitutes	395(78.4)	72(18.2)	1.00		--	--	--	
Always	34(6.7)	8(23.5)	1.46	(0.63-3.41)	--	--	--	--
Not always	75(14.9)	23(30.7)	1.78	(1.00-3.17)	--	--	--	--
*P-trend*				0.0128				

^a^Due to missing data, all categories do not total 504.

^b^Odds ratio and 95% confidence intervals obtained using logistic regression models adjusted for age and SLI.

^c^Models adjusted for SLI only to avoid colinearity.

^d^Models adjusted for age only to avoid colinearity when adjusting for SLI.

^e^This variable as was asked of men only. Women were assigned the value corresponding to the antecedent of circumcision in their male sexual partner.

**Table 3:** Risk of HPV infection associated with the status of HPV infection in the sexual partner

Variable	Risk of HPV infection in women				
*Presence of HPV* *in men*	n = 504	HPV positives n = 69%	**OR**^**a**^	**ρ**^**a**^	**CI 95%**^**a**^
**Presence of HPV**					
Negative	401/79.6	8.7(35)			
Positive	103/20.4	33.0(34)	5.15	0.000	3.01 - 8.82
**Presence of oncogenic HPV**					
Negative	460	6.9(32)			
Positive	44	36.4(16)	7.64	0.000	3.75 - 15.56
**Presence of nononcogenic HPV**					
Negative	429	3.7(16)			
Positive	75	22.7(17)	7.56	0.000	3.62 - 15.79
**Presence of HPV**					
16 and/or 18					
Negative	491	2.4(12)			
Positive	13	15.4(2)	7.25	0.016	1.44 - 36.37

^a^Odds ratio, p-value, and CI 95% obtained using logistic regression.

We are thankful for your observations and deeply regret the confusion in the results presented.

## Pre-publication history

The pre-publication history for this paper can be accessed here:

http://www.biomedcentral.com/1471-2334/11/25/prepub
